# Nicotinamide attenuates the decrease in dendritic spine density in hippocampal primary neurons from 5xFAD mice, an Alzheimer’s disease animal model

**DOI:** 10.1186/s13041-020-0565-x

**Published:** 2020-02-07

**Authors:** Hyunju Kim, Bora Kim, Hye-Sun Kim, Joo-Youn Cho

**Affiliations:** 1 Department of Pharmacology, College of Medicine, Seoul National University, 103 Daehakro, Jongro-gu, Seoul, Republic of Korea; 2 Department of Biomedical Sciences, College of Medicine, Seoul National University, 103 Daehakro, Jongro-gu, Seoul, Republic of Korea; 3 Department of Clinical Pharmacology and Therapeutics, College of Medicine, Seoul National University, 103 Daehakro, Jongro-gu, Seoul, Republic of Korea; 4 Kidney Research Institute, College of Medicine, Seoul National University, 103 Daehakro, Jongro-gu, Seoul, Republic of Korea; 5 Seoul National University College of Medicine, Bundang Hospital, Bundang-Gu, Sungnam, Republic of Korea; 6 Department of Pharmacology and Biomedical Sciences, Neuroscience Research Institute, College of Medicine, Seoul National University, 103 Daehakro, Jongro-gu, Seoul, Republic of Korea; 7 Department of Clinical Pharmacology and Therapeutics, Seoul National University College of Medicine and Hospital, 101 Daehak-ro, Jongno-gu, Seoul, 03080 Republic of Korea

**Keywords:** Alzheimer’s disease, Dendritic spine density, Hippocampus, Metabolomics, Nicotinamide

## Abstract

Alzheimer’s disease (AD) is the most common neurodegenerative disease characterized by memory loss and the presence of amyloid plaques and neurofibrillary tangles in the patients’ brains. In this study, we investigated the alterations in metabolite profiles of the hippocampal tissues from 6, 8, and 12 month-old wild-type (WT) and 5xfamiliar AD (5xFAD) mice, an AD mouse model harboring 5 early-onset familiar AD mutations, which shows memory loss from approximately 5 months of age, by exploiting the untargeted metabolomics profiling. We found that nicotinamide and adenosine monophosphate levels have been significantly decreased while lysophosphatidylcholine (LysoPC) (16:0), LysoPC (18:0), and lysophosphatidylethanolamine (LysoPE) (16:0) levels have been significantly increased in the hippocampi from 5xFAD mice at 8 months or 12 months of age, compared to those from age-matched wild-type mice. In the present study, we focused on the role of nicotinamide and examined if replenishment of nicotinamide exerts attenuating effects on the reduction in dendritic spine density in hippocampal primary neurons from 5xFAD mice. Treatment with nicotinamide attenuated the deficits in spine density in the hippocampal primary neurons derived from 5xFAD mice, indicating a potential role of nicotinamide in the pathogenesis of AD. Taken together, these findings suggest that the decreased hippocampal nicotinamide level could be linked with AD pathogenesis and be a useful therapeutic target for AD.

## Introduction

Alzheimer’s disease (AD) animal models have been used to investigate pathogenic mechanisms, discover potential biomarkers, and evaluate novel treatments [1, 2]. The 5xFAD mouse, a widely used AD mouse model, has 5 familiar AD-linked mutations, i.e., the Swedish (K670 N/M671 L), Florida (I716V), and London (V717I) mutations in amyloid precursor protein (APP) and the M146 L and L286 V mutations in presenilin-1 (PSEN1) [2]. 5xFAD mice, characterized by cerebral amyloid plaques and gliosis, show massive Aβ_1–42_ burdens from 2 months of age, declined synaptic markers from 4 months of age, and cognitive impairment from approximately 5 months of age [2–5]. Thus, pathological mechanisms of AD could be evaluated by analyzing biochemical changes in the brain in 5xFAD mice at different disease stages.

Altered metabolites reflect downstream changes of genomic, transcriptomic, and proteomic fluctuations, and metabolomics data, such as an accurate biochemical profile, can thus be used to visualize and interpret complex biological networks of AD. Multiple human studies have focused on metabolomics analysis of blood-derived samples, cerebrospinal fluid, and postmortem brain tissues, since, unlike for mouse models, premortem brain samples are not available [6]. Targeted metabolomics of 44 postmortem brain samples showed that a panel of sphingolipids is associated with the severity of AD pathology [7–9]. Arginine metabolism is altered in the postmortem hippocampus, superior frontal gyrus, and cerebellum of AD patients and normal control subjects [10].

Brain metabolic perturbations have been described in several transgenic AD models. For instance, the disturbances in metabolites of the glycolytic pathway (glucose-6-phosphate and glycerol-3-phosphate) and tricarboxylic acid (TCA) cycle (α-ketoglutarate, fumarate, and succinate) were identified in astrocytes derived from newborn 5xFAD mice [11], and pantethine treatment reduced the extent of metabolic perturbation and decreased the inflammatory processes in these astrocytes, indicating the role of altered brain energetics in the AD pathogenesis; metabolic profile analyses revealed region-specific metabolic changes in the hippocampus, cortex, cerebellum, and olfactory bulbs in APP/PS1 mice [12, 13], and metabolomics signatures, including mitochondrial dysfunction and altered energy metabolism indicated by changes in nucleotide, TCA cycle, energy transfer, neurotransmitter, and amino acid metabolic pathways, were identified in APP/PS1 mice [14]; in addition, significant changes in metabolite compositions, including accumulation of fatty acids, alterations in phospholipids and acylcarnitines related to neural membrane degradation, and impaired energy management, were observed in the hippocampus and cortex in APP/PS1 mice [13]. Because the metabolic pathways are conserved through evolution [15, 16], the metabolic signatures identified in AD mouse models could be directly translated into human studies [17]. Therefore, metabolomics screening in transgenic models could be useful for the understanding of the pathological mechanisms of AD.

Amyloid β peptide (Aβ) deposition and neurofibrillary tangles in the AD hippocampus, which is the central brain region to exhibit neurodegeneration and other AD-related alterations, possibly lead to cognitive impairment [18, 19]. In addition, hippocampal oxidative stress is implicated in neurodegenerative diseases and neurodevelopmental disorders [20, 21]. However, no study has investigated the metabolomics profiling of the hippocampus in the 5xFAD mouse model. This study aims to apply an untargeted metabolomics approach to characterize metabolic abnormalities in the hippocampus in 5xFAD mice at different AD progression stages.

## Materials and methods

### Experimental animals

All experimental procedures were approved by the Animal Care Committee of Seoul National University (Approval Number: SNU-131016-1). Transgenic mice with 5 familial AD mutations were purchased from Jackson Laboratories (strain: B6SJL-Tg [APPSwFlLon, PS1*M146 L*L286 V] 6799Vas/J) and were bred by crossing hemizygous transgenic male mice with B6SJL F1 female mice. Male WT and 5xFAD mice were used in all experiments. Animal treatment and maintenance were performed in accordance with the Institutional Animal Care and Use Committee Guidelines of Seoul National University, Seoul, Korea.

### Chemicals and reagents

High-performance liquid chromatography grade solvents, including methanol, acetonitrile, and water, were purchased from J.T. Baker (PA, USA). Formic acid, nicotinamide, adenosine monophosphate, LysoPC, and LysoPE were obtained from Sigma-Aldrich (MO, USA) and Avanti Polar Lipids (AL, USA).

### Hippocampus sample preparation for metabolomics

The hippocampus was weighed (~ 20 mg wet), homogenized in methanol: water (4:1, v/v; 50 μl/mg tissue), and frozen in liquid nitrogen for 1 min. The homogenate was thawed at room temperature and was then sonicated for 5 s. After adding acetonitrile (30 μl/mg tissue), the homogenate was vortexed for 5 s incubated for 1 h at − 20 °C, and centrifuged at 13,000 rpm for 15 min at 4 °C. The pellet was reconstituted in radioimmunoprecipitation assay buffer (Elpis-Biotech, Daejeon, Korea), and the concentration of total protein was determined using the Pierce BCA Protein Assay Kit (Thermo Scientific, MA, USA). The supernatant was transferred into a microcentrifuge tube and was dried under an N2 evaporator. The dry extracts were then reconstituted with different volumes of solvent mixtures (acetonitrile: H_2_O, 1:1, v/v) based on the sample’s protein levels, and the mixtures were sonicated for 10 min and centrifuged at 14,000 rpm for 15 min at 4 °C to remove insoluble debris. The supernatant was used for the LC-MS analysis.

### Untargeted metabolomics

A 4 μl aliquot of the sample was injected into a Waters UPLC system with a reverse phase 2.1 × 100 mm ACQUITY 1.8 μm HSS T3 column. The gradient mobile phase comprised 0.1% formic acid (Solution A) and methanol containing 0.1% formic acid (Solution B). Each sample was resolved for 20 min at a flow rate of 0.4 ml/min. The gradient consisted of 5% Solution B for 1 min, 5–30% Solution B over 1–8 min, 30–70% Solution B over 8–13 min, and 95% Solution B for 14 min (maintaining for 2 min). The samples were equilibrated in 95% Solution A for 3.5 min before injection. A Waters Xevo G2 time-of-flight mass spectrometry was operated in positive and negative ionization modes. To obtain consistent differential variables, we prepared a pooled sample (quality control [QC] sample) by mixing aliquots of individual samples. Replicates of the QC sample were acquired in a series of injections, and data were obtained by random injection. The metabolomic dataset was deconvoluted and peak aligned using Progenesis QI software (version 2.3, Nonlinear Dynamics, Newcastle, UK). The most suitable candidate QC sample was chosen by the highest similarity using Progenesis QI software. Vector alignment quality was manually processed, and files were aligned with sensitivity (10 ppm), retention time limits, and peak normalization (normalization to all compounds) at the default values. Subsequently, ions with a % CV of abundance > 30 in the QC were removed. A significantly differential expression was defined as a false discovery rate (FDR) adjusted *p*-value (*q*-value) < 0.05. The FDR was obtained by adjusting the raw *p*-values of the *t*-test using the method of Benjamini and Hochberg [22].

### Hippocampal primary neuron culture

Hippocampal primary neurons, which were prepared from postnatal day 1 to 2 5xFAD mice by dissociating with 0.25% trypsin, were plated onto coverslips coated with poly-L-lysine (Sigma, St Louis, USA). Neurons were grown in Neurobasal medium (Gibco, CA, USA) supplemented with B27 (Gibco, CA, USA), 2 mM GlutaMAX-I (Gibco, CA, USA), and 100 μg/ml penicillin/streptomycin (Gibco, CA, USA) at 37 °C in a humidified environment of 95% O_2_/5% CO_2_.

### Dendritic spine density analysis

Hippocampal primary neurons were transfected with 6 μg CAG-IRES-mGFP plasmid (a generous gift from Dr. Kolodkin) in 18-mm glass coverslips in 60-mm dishes. Nicotinamide (Sigma, St Louis, USA) was prepared in saline. Neurons were treated with nicotinamide or vehicle for 24 h. The number of dendritic spines was evaluated at 18–19 days in vitro (DIV). Fluorescent images were acquired using a confocal microscope (LSM 510; Carl Zeiss, Jena, Germany) using the same setting condition for all samples. Spines were counted within the 20-μm to 50-μm segments on secondary dendrites extending 50–100 μm beyond the soma.

### Statistical analysis

One-way ANOVA and independent *t*-test (SPSS, IL, USA) were used for determining the statistical significance. A *p* < 0.05 or *q* < 0.05 was considered statistical significance.

## Results

### Hippocampus metabolic profiling

**Fig. 1:**
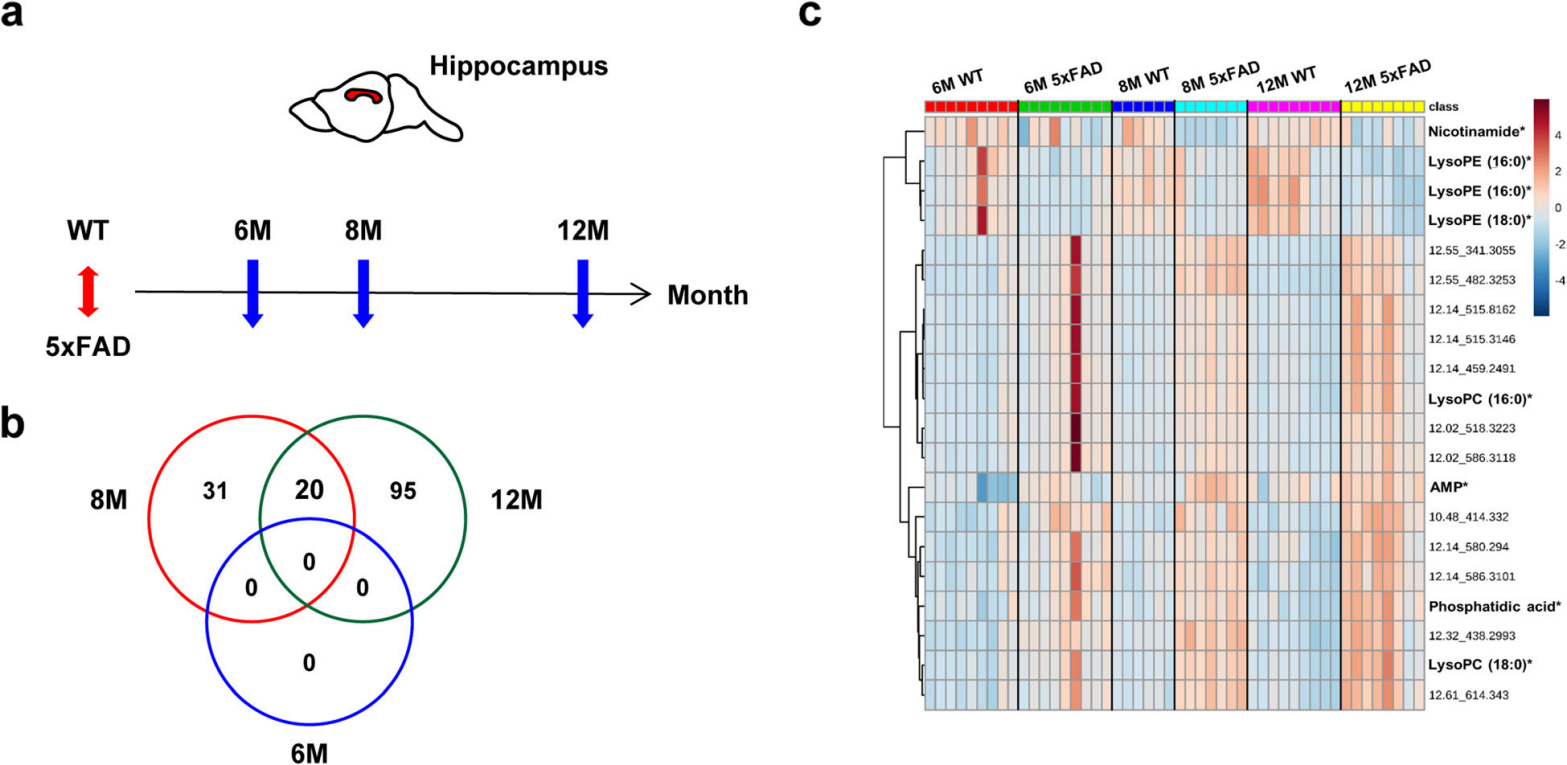
Hippocampal metabolomics of 5xFAD mice at different disease progression stages. **a** Time course of the hippocampal sample collection. **b** Venn diagram representing overlapping ion features that were significantly different between the hippocampi of WT and 5xFAD mice (*q* < 0.05) at 6, 8, or 12 months of age. **c** Hierarchically clustered heat map of the relative intensity of 20 metabolic markers. Rows and columns represent the individual mice and the 20 selected metabolites (retention time_m/z, *identified or putative metabolites), respectively. Each cell is colored based on the relative intensity

**Fig. 2:**
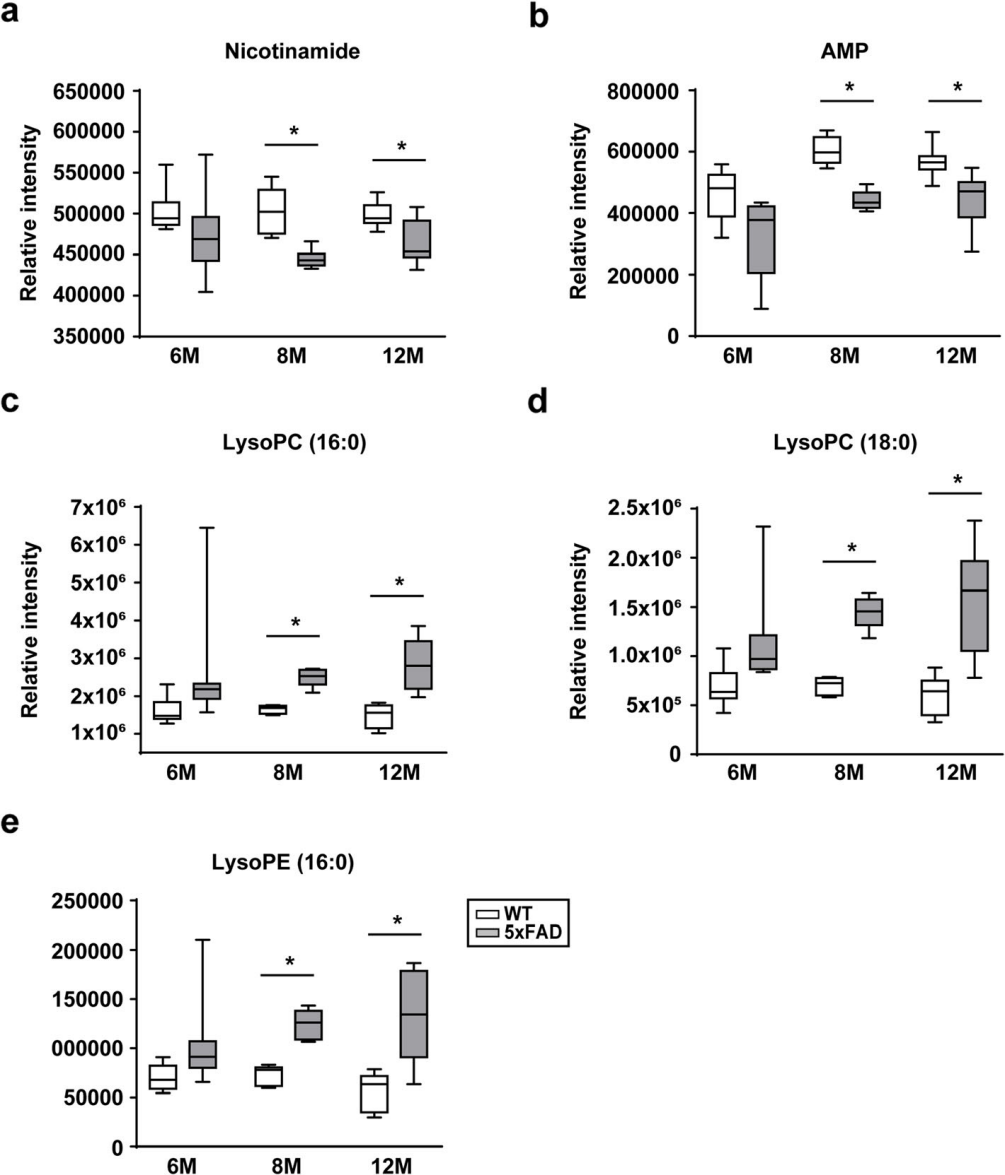
Relative abundance of metabolites in the hippocampus in 5xFAD mice at each age. **a** nicotinamide, **b** AMP, **c** LysoPC (16:0), **d** LysoPC (18:0), and **e** LysoPE (16:0). Data are shown as means ± SEM of at least 3 independent experiments; 6 M-WT (*n* = 9), 6 M-5xFAD (*n* = 9), 8 M-WT (*n* = 6), 8 M-5xFAD (*n* = 7), 12 M-WT (*n* = 9), and 12 M-5xFAD (*n* = 8). *q < 0.05 compared with age-matched WT mice. AMP, adenosine monophosphate; LysoPC, lysophosphatidylcholine; LysoPE, lysophosphatidylethanolamine

Untargeted metabolomics profiling of hippocampal tissues was performed in WT and 5xFAD mice at three different stages (6, 8, and 12 months) (Fig. 1 a). The number of WT and 5xFAD mice and the weights of hippocampal tissues used are described in Table 1. In total, 2950 compound ions were identified in the positive ion and negative ion electrospray ionization (ESI^+^ and ESI^−^, respectively) modes. We selected ions with a *q*-value of less than 0.05 in 5xFAD mice at each stage. Notably, 51 and 115 features were significantly different between WT and 5xFAD mice at 8 and 12 months of age, respectively (Fig. 1b). No markers differed between WT and 5xFAD mice at 6 months of age. Twenty ions that were significantly different between WT and 5xFAD mice at both 8 and 12 months of age were selected for further identification. The hierarchically clustered heat map illustrates significant differences in the relative intensity of the selected 20 markers (Fig. 1c). The selected ions are described in Table 2**.** After removing the ion-source fragment features, 5 metabolites were identified as nicotinamide, adenosine monophosphate, LysoPC (16:0), LysoPC (18:0), and LysoPE (16:0) by comparing the MS/MS spectrum of each metabolite with that of the authentic compound. The levels of nicotinamide and adenosine monophosphate were significantly lower in 5xFAD mice than in WT mice (Fig. 2a and b), whereas the levels of LysoPC (16:0), LysoPC (18:0), and LysoPE (16:0) were significantly higher (*q* < 0.05) in 5xFAD mice than in WT mice at 8 or 12 months of age (Fig. 2c, d, and e).

**Table 1:** The number of male mice and tissue weights. The mice were classified into 6 groups based on age (6, 8, and 12 months). HPC, hippocampus; SD, standard deviation

Age (month)	Genotype	HPC weight (mg)	*N*
		Mean ± SD	
		(min-max)	
6	WT	20.5 ± 3.4	9
		(15.0–25.6)	
	5xFAD	20.3 ± 5.4	9
		(14.5–28.6)	
8	WT	21.1 ± 2.7	6
		(17.8–25.7)	
	5xFAD	24.4 ± 3.2	7
		(20.4–29.2)	
12	WT	19.7 ± 4.2	9
		(10.0–24.9)	
	5xFAD	18.4 ± 2.8	8
		(13.1–21.8)

**Table 2:** Hippocampal biomarkers that were significantly different (*q* < 0.05) both in 8 and 12 months

Metabolites	Regulation^a^	8 M		12 M		Mass	RT	Ion mode	Fragment ion (m/z)
		*q-value*	*p-value*	*q-value*	*p-value*	(m/z)	(min)		
Nicotinamide^b^	down	0.00161	0.00044	0.00161	0.00044	123.0554	0.97	POS	80.0502, 78.0339, 96.0439
LysoPC (16:0)^b^	up	1.62E-05	1.52E-06	1.62E-05	1.52E-06	496.3402	12.14	POS	184.0735, 104.1076
LysoPC (18:0)^b^	up	5.07E-07	1.09E-08	5.07E-07	1.09E-08	524.3719	12.61	POS	184.0739, 104.1074
Phosphatidic acid^c^	up	2.01E-05	2.22E-06	2.01E-05	2.22E-06	487.2795	12.61	POS	445.2678
AMP^a^	down	4.10E-06	8.02E-07	4.10E-06	8.02E-07	346.0545	0.97	NEG	96.9692, 134.0466, 150.9503
LysoPE (16:0)^c^	up	1.67E-07	2.10E-08	1.67E-07	2.10E-08	452.2761	11.98	NEG	255.2324, 112.9855, 196.0417
LysoPE (16:0)^a^	up	5.52E-08	5.39E-09	5.52E-08	5.39E-09	452.2772	12.1	NEG	255.2326, 122.9857, 196.0413
LysoPE (18:0)^c^	up	1.37E-06	2.20E-07	1.37E-06	2.20E-07	480.3085	12.13	NEG	255.2322

^a^Up- or down- regulation in 5xFAD vs. WT. ^b^ identified metabolite compared with authentic standard. ^c^putative metabolites compared with database. *RT* Retention time, *CE* Colision energy

### Nicotinamide supplementation rescues the spine deficits in hippocampal primary neurons derived from 5xFAD mice

Dendritic spine alteration is the cellular mechanism underlying neuronal activity and memory. It has been reported that treatment with nicotinamide rescues both short- and long-term memory impairment in 3xTg-AD mice, the triple-transgenic mice which harbor a knock-in mutation of PSEN1_M146V_, the Swedish double mutation of APP_KM670/671NL_, and a frontotemporal dementia mutation in tau (tau_P301L_) on a 129/C57BL/6 background [23].

**Fig. 3:**
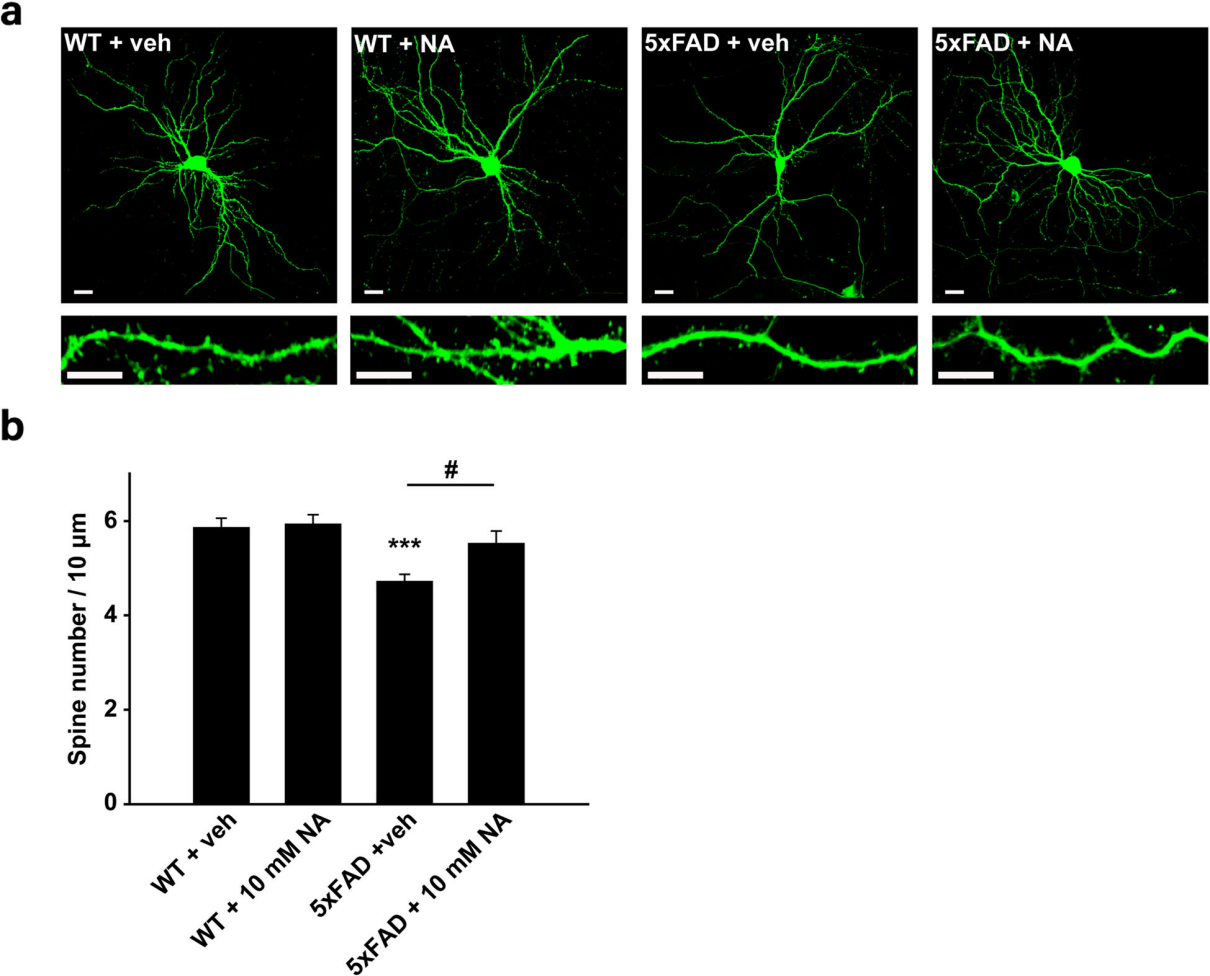
Nicotinamide treatment rescues synaptic loss in hippocampal primary neurons derived from 5xFAD mice. **a** Representative images of dendritic spines in primary WT and 5xFAD hippocampal neurons at 18–19 DIV. The dendritic segment outlined with a white box (upper) is magnified to delineate the spine morphology (bottom) with a 3× optic zoom. The scale bars indicate 20 μm and 10 μm in the low- and high-magnification images, respectively. **b** Quantification of the spine densities. The dendritic spine densities were significantly reduced in neurons derived from 5xFAD mice (*n* = 28 neurons, one-way ANOVA) compared with those from WT mice (*n* = 39 neurons). Treatment with nicotinamide significantly attenuated the reduction in dendritic spine density in primary hippocampal neurons derived from 5xFAD mice (*n* = 17 neurons, one-way ANOVA). Data are represented as mean ± SEM. ^*^p < 0.05, ^***^p < 0.001 compared with mGFP-transfected WT hippocampal primary neurons; ^#^*p* < 0.001 compared with mGFP-transfected 5xFAD hippocampal primary neurons. NA, nicotinamide; DIV, days in vitro

Dendritic spine density in hippocampal primary neurons from 5xFAD mice was found to be significantly lower than hippocampal primary neurons from WT mice (WT + vehicle [*n* = 4], 5.84 ± 0.206 [spine number/μm]; 5xFAD + vehicle [*n* = 3], 4.71 ± 0.160 [spine number/μm], *p* < 0.001) (Fig. 3a and b). Next, we examined whether treatment with nicotinamide rescues the decrease in dendritic spine density in hippocampal primary neurons cultured from 5xFAD mice. Treatment with 10 mM nicotinamide for 24 h rescued the reduction in dendritic spine density in hippocampal primary neurons at 17–18 DIV (5xFAD + vehicle [n = 3], 4.71 ± 0.160 [spine number/μm]; 5xFAD + 10 mM nicotinamide [n = 3], 5.52 ± 0.270 [spine number/μm], *p* < 0.05), while the same treatment did not affect dendritic spine density of WT hippocampal neurons (WT + vehicle [*n* = 4], 5.84 ± 0.206 [spine number/μm]; WT + 10 mM nicotinamide [n = 4], 5.93 ± 0.202 [spine number/μm]) (Fig. 3a and b). These findings indicate that reduced dendritic spine density in hippocampal primary neurons is, at least in part, due to the decreased nicotinamide concentration in the hippocampi from AD brains.

## Discussion

AD is usually clinically diagnosed after its pathophysiological process has already started. In the early AD stage, patients show mild cognitive impairment, which develops into AD in the rate of approximately 15% per year [24, 25]. Therefore, the understanding of molecular pathogenesis in the pre-clinical process is essential for identifying treatment targets.

While there are some reports that have determined the metabolomics profiles associated with the progression of AD using postmortem brain tissues and blood samples [7, 26, 27], we are the first to address alterations in brain metabolism associated with AD in the hippocampus of 5xFAD mice in three different disease stages.

Nicotinamide, an amide form of vitamin B3, is the primary precursor of nicotinamide adenine dinucleotide (NAD^+^) in mammalian cells [28, 29]. Nicotinamide is converted to NAD^+^ through the activity of nicotinamide phosphoribosyltransferase, a rate-limiting enzyme in NAD^+^ biosynthesis. As an energy substrate and cofactor for many enzymes, NAD^+^ is critical for mitochondrial health and neuronal stress resistance [30, 31]. NAD^+^ is a crucial cofactor for cellular processes, such as glycolysis, fatty acid β-oxidation, tricarboxylic acid cycle, and DNA repair [32, 33]. NAD^+^-dependent signaling, which is associated with neuronal development, survival, and function in the central nervous system, is implicated in neuroprotection [34]. The potential role of nicotinamide in AD has been highlighted in several studies. For instance, oral treatment with nicotinamide improves cognitive performance and reduces Aβ and hyperphosphorylated tau pathologies in 3xTg-AD mice [34], and nicotinamide preserves cellular NAD^+^ levels and increases the resistance of neurons against excitotoxicity [35]. The findings indicate the neuroprotective effect of nicotinamide and NAD^+^. However, no study has investigated the alterations of nicotinamide in the brain of AD models or patients. Although many reports have demonstrated the potential role of nicotinamide in neuroprotection and cognition, how the nicotinamide level changes in AD is not known. Here, we report novel findings that nicotinamide levels are decreased in the hippocampus of 5xFAD mice. This finding supports the hypothesis that NAD + -dependent signaling is disturbed in the AD brain.

In this study, we have investigated whether the supplementation of nicotinamide attenuates the reduction in dendritic spine density using cultured primary neurons from the hippocampus of 5XFAD mice (Fig. 3). Recent evidence has strongly indicated that cultured neurons from AD mouse models represent valuable models of this neurodegenerative disorder. Neurons derived from Tg2576 mice, a mouse model carrying a single mutation in human APP, show high intensity staining for the human APP protein/Aβ fragments and increased vulnerability [36]. Additionally, the total number of dendritic spines, total spine extent, spine surface area, spine head diameter, and spine cross-sectional area are significantly decreased in neurons from APP/PS1 mice, a mouse model carrying double AD-associated mutations [37]. Synaptic loss is one of the pathological hallmarks of AD and best correlates with cognitive decline, suggesting that it is a critical event in the pathophysiology of the disease [38]. Based on these previous reports, it can be said that cultured neurons from AD animal model reflect AD phenotypes in vitro and can be used in AD study. Thus, we assumed that the primary neurons from 5xFAD reflect the hippocampal pathophysiological characteristics of 5xFAD and treated nicotinamide to the primary neurons. Treatment with nicotinamide rescued synaptic deficits in hippocampal primary neurons derived from 5xFAD mice (Fig. 3). Together with those in the study by Liu et al. [34], our findings indicate that nicotinamide is a potential therapeutic drug for AD. However, further studies are needed to investigate whether nicotinamide supplementation improves cognition in 5xFAD mice.

Reduced adenosine monophosphate levels have significant consequences, such as impairments in cellular energy homeostasis, because adenosine monophosphate plays a central role in glucose and lipid metabolism through the adenosine monophosphate-activated protein kinase, which is known to be decreased in AD brains [39, 40]. In addition, significantly decreased adenosine monophosphate levels were detected in the hippocampus and cortex of APP/PS1 mice [13]. Notably, elevated adenosine monophosphate deaminase activity, which has been identified in the postmortem brain in AD patients, might lead to degradation of adenosine monophosphate and over-production of ammonia [41].

We found that lysoPC and lysoPE levels increased in the hippocampus in 5xFAD mice. Phospholipids, including lysoPC and lysoPE, are metabolized by phospholipase A_2_ (PLA_2_) from PC and PE, respectively [42]. PLA_2_ activity has been shown to vary in different AD stages. In the early AD stage, PLA_2_ activity is decreased in the brains of AD patients [43, 44]. However, as the disease advances, PLA_2_ activity is elevated in AD brains [45]. Increased activation of PLA_2_ might induce an inflammatory condition by activating the arachidonic acid cascade, which plays a vital role in the inflammatory process. Furthermore, it has been demonstrated that LysoPC induces the formation of oligomer Aβ and subsequent neurodegeneration in cultured neuronal cells [46]. Therefore, increased phospholipid levels might be associated with elevated PLA_2_ activity and increased inflammation in AD brains.

## Conclusion

**Fig. 4:**
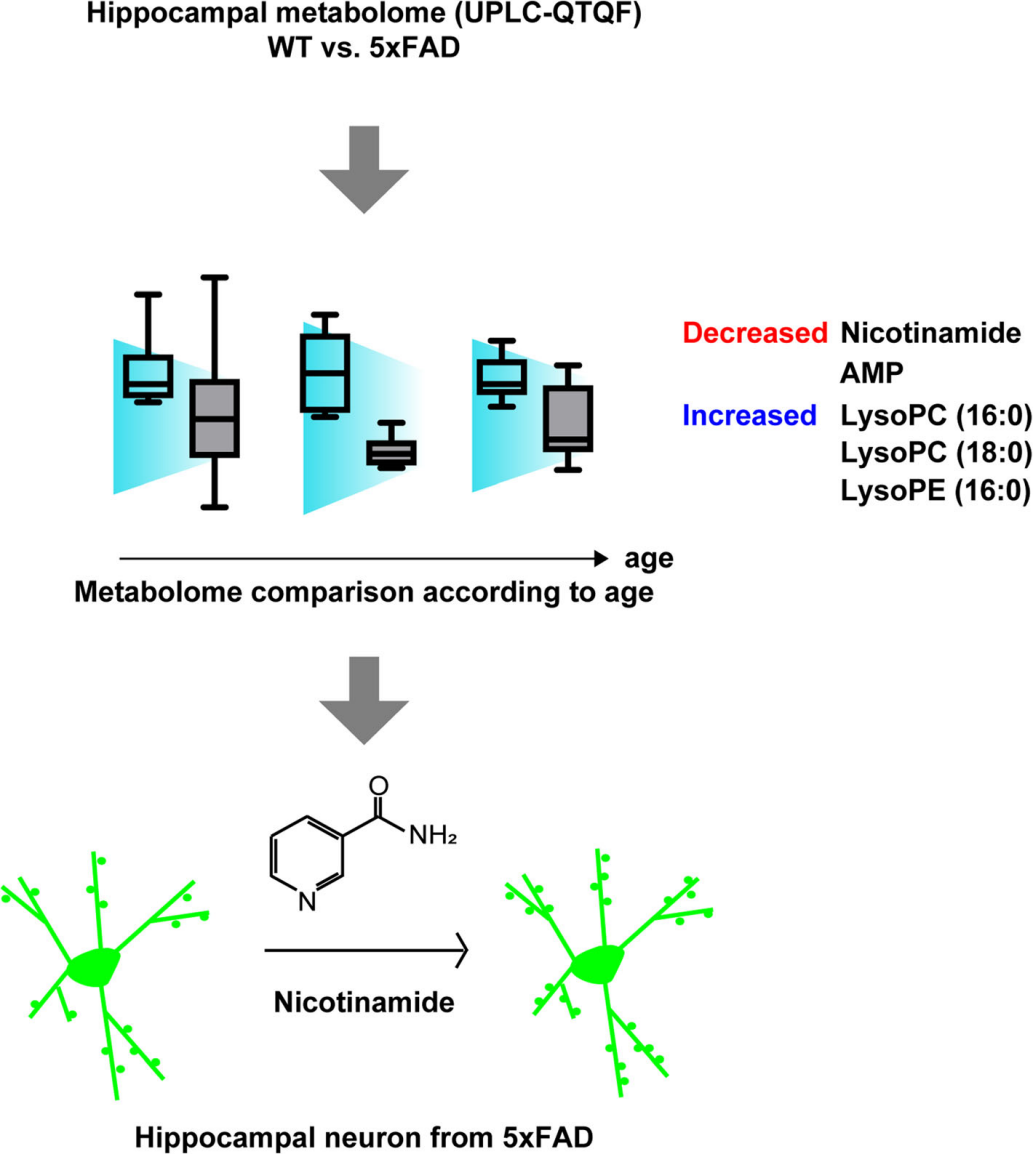
A graphical summary of this study, i.e. the reduction in nicotinamide in the hippocampus from 5xFAD mice as evaluated with untargeted metabolomics profiling, and the effect of supplementation on dendritic spine density of hippocampal primary neuron cultures from 5xFAD mice. 5xFAD mice displayed decreased content of nicotinamide and AMP, and increased content of LysoPC (16:0), LysoPC (18:0), LysoPE (16:0) in the hippocampus age-dependently. The addition of nicotinamide to hippocampal primary neuron culture from 5xFAD mice restored the reduced dendritic spine density. These results suggest nicotinamide as a therapeutic target in AD

This study is the first to investigate hippocampal metabolic markers in 5xFAD mice using an untargeted metabolomics approach. Another important aspect of this work is the comprehensive analysis across different disease progression stages. As summarized in the graphical summary of Fig. 4, we found decreased nicotinamide and adenosine monophosphate levels and increased LysoPC (16:0), LysoPC (18:0), and LysoPE (16:0) levels in the hippocampi in 5xFAD mice at 8 or 12 months of age. We also demonstrated that nicotinamide rescued the synaptic deficits of 5xFAD hippocampal primary neurons. This study is the first to demonstrate the reduced hippocampal nicotinamide levels in 5xFAD mice, and the findings suggest that the hippocampal nicotinamide level could be a useful therapeutic target for AD. Further studies are needed to clarify the metabolic pathway of nicotinamide and the molecular mechanism underlying altered nicotinamide levels in the hippocampus of 5xFAD mice.

## Data Availability

Not applicable.

## Abbreviations

5xFAD: Five human familial AD
AD: Alzheimer’s disease
APP: Amyloid precursor protein
Aβ: Amyloid β peptide
DIV: Days in vitro
ESI: Electrospray ionization
FDR: False discovery rate
Lyso PC: Lysophosphatidylcholine
Lyso PE: Lysophosphatidylethanolamine
NAD^+^: Nicotinamide adenine dinucleotide
PLA_2_: Phospholipase A_2_
PSEN 1: Presenilin-1
QC: Quality control
TCA: Tricarboxylic acid
WT: Wild type
